# Early Osteogenic-Induced Adipose-Derived Stem Cells and Canine Bone Regeneration Potential Analyzed Using Biodegradable Scaffolds

**DOI:** 10.3390/bioengineering10111311

**Published:** 2023-11-13

**Authors:** Hyun-Ho Yun, Seong-Gon Kim, Se-Il Park, Woori Jo, Kyung-Ku Kang, Eun-Joo Lee, Dong-Kyu Kim, Hoe-Su Jung, Ji-Yoon Son, Jae-Min Park, Hyun-Sook Park, Sunray Lee, Hong-In Shin, Il-Hwa Hong, Kyu-Shik Jeong

**Affiliations:** 1 Department of Veterinary Pathology, College of Veterinary Medicine, Kyungpook National University, Daegu 41566, Republic of Korea; hyun6551@kmedihub.re.kr (H.-H.Y.); qzpmqzpm@naver.com (K.-K.K.); miffy525@hanmail.net (E.-J.L.); jiyoon1095@naver.com (J.-Y.S.); kow612@naver.com (J.-M.P.); 2 Preclinical Research Center, Daegu-Gyeongbuk Medical Innovation Foundation, Daegu 41061, Republic of Korea; sgkim@kmedihub.re.kr (S.-G.K.); c2dar@kmedihub.re.kr (W.J.); dgkim728@kmedihub.re.kr (D.-K.K.); junghs2000@kmedihub.re.kr (H.-S.J.); 3 Cardiovascular Product Evaluation Center, Yonsei University College of Medicine, Seoul 03722, Republic of Korea; seil-park@hanmail.net; 4 Cell Engineering for Origin Research Center, Seoul 03150, Republic of Korea; hsparkkwon@hotmail.com (H.-S.P.); sunray@cefobio.com (S.L.); 5 Department of Oral Pathology and Regenerative Medicine, School of Dentistry, Kyungpook National University, Daegu 41940, Republic of Korea; hishin@knu.ac.kr; 6 Department of Veterinary Pathology, College of Veterinary Medicine, Gyeongsang National University, Jinju 52828, Republic of Korea; ihhong@gnu.ac.kr; 7 Institute for Next Generation Unified Technology, Hoseo University, Asan 31499, Republic of Korea

**Keywords:** canine adipose-derived mesenchymal stem cells, adipose-derived stem cells, osteodifferentiation

## Abstract

The complex process of bone regeneration is influenced by factors such as inflammatory responses, tissue interactions, and progenitor cells. Currently, multiple traumas can interfere with fracture healing, causing the prolonging or failure of healing. In these cases, bone grafting is the most effective treatment. However, there are several drawbacks, such as morbidity at the donor site and availability of suitable materials. Advantages have been provided in this field by a variety of stem cell types. Adipose-derived stem cells (ASCs) show promise. In the radiological examination of this study, it was confirmed that the C/S group showed faster regeneration than the other groups, and Micro-CT also showed that the degree of bone formation in the defect area was highest in the C/S group. Compared to the control group, the change in cortical bone area in the defect area decreased in the sham group (0.874), while it slightly increased in the C/S group (1.027). An increase in relative vascularity indicates a decrease in overall bone density, but a weak depression filled with fibrous tissue was observed outside the compact bone. It was confirmed that newly formed cortical bone showed a slight difference in bone density compared to surrounding normal bone tissue due to increased distribution of cortical bone. In this study, we investigated the effect of bone regeneration by ADMSCs measured by radiation and pathological effects. These data can ultimately be applied to humans with important clinical applications in various bone diseases, regenerative, and early stages of formative differentiation.

## 1. Introduction

Bone is a complex tissue that participates not only in body movement, but also in several physiological processes, such as mineral (calcium and phosphate) homeostasis and storage, endocrine function, and hematopoiesis in the bone marrow. Bone fractures and defects can be life-threatening injuries, owing to the slow nature of healing. The global issue of bone fractures, particularly prevalent among individuals with osteoporosis, not only poses a significant public health concern but also imposes substantial economic burdens. Fractures contribute to widespread work absenteeism, reduced productivity, disability, diminished quality of life, health deterioration, and substantial healthcare expenses, creating a substantial strain on individuals, families, communities, and healthcare systems. A comprehensive meta-analysis of existing studies revealed that the aggregated cost for hospital-based treatment of a hip fracture was estimated at USD 10,075. Furthermore, the overall health and social care expenses for a single hip fracture over a span of 12 months were calculated to be an average of USD 43,669.7 globally [1]. Serious cases, such as nonunion, can cause disability and reduce quality of life. Restoring large segmental defects in the bone is challenging for veterinarians. Classic therapeutic standards use autologous bone grafts or allografts [1], which are associated with substantial morbidity, including infection, disease transmission, and loss of function [2,3,4]. Under certain circumstances, bone grafts have the potential to result in the formation of scar tissue, necessitating a subsequent iteration of the bone graft procedure to promote proper bone growth at the intended site. There are instances where the body may reject foreign materials, or the procedure itself could inadvertently harm adjacent structures such as neighboring teeth, nerves, or blood vessels, underscoring the need for careful consideration and monitoring during these medical interventions. In addition, the utilization of cancellous bone materials and bone substitute materials incurs comparable direct costs, with potential variations in smaller components, particularly if scoring techniques are applied, owing to the elevated failure rates associated with these smaller parts [2]. New therapeutic strategies for non-union healing have been studied for several decades and various treatments have been developed to improve fracture healing prognosis and healing rates. Strategies to overcome these drawbacks include synthesis or allogenic scaffolding with implantation of cells for bone regeneration [4,5].

Cell therapy typically involves the injection, grafting, and implantation of viable cells into damaged tissues to induce regeneration. Stem cell transplantation has consistently attracted attention as a potential technology for various degenerative and immunopathological disease states. Adult stem cells are usually used as treatments because they tend to be therapeutically stable and safe, unlike embryonic stem cells (ESCs), which have ethical limitations, and induced pluripotent stem cells (iPSCs), which have tumor risks. There are three types of adult stem cells: hematopoietic stem cells (HSCs), mesenchymal stem cells (MSCs), and neural stem cells (NSCs). MSCs are primarily used to treat skeletal diseases [6]. Our research team has previously used MSCs for skeletal therapy for horses and fracture and osteoarthritis models, demonstrating significant improvements [7].

MSCs can be extracted from bone marrow, fatty tissue, cord blood, and synovium. For the past 30 years, bone-marrow-derived mesenchymal stem cells (BMMSCs) have been a popular cellular source for regenerative medicine research. However, the BMMSC isolation procedure is difficult on the donors and patients, and the number of stem cells extracted is low. Adipose tissue has recently been identified as an alternative source of multipotent stem cells. As the body has abundant adipose tissue, these resident stem cells that differentiate into multiple mesenchymal streaks are easily collected. Adipose-derived mesenchymal stem cells (ADMSCs) show better bone treatment outcomes than BMMSCs [8,9]. ADMSCs improve bone formation and angiogenesis, which accelerates fracture healing. These cells can differentiate into adipocytes, chondrocytes, osteoblasts, fibroblasts, and endothelial cells [10,11,12]. ADMSCs have also been studied as a cell source for transplantation into scaffolds for bone regeneration [13,14]. As a source of autologous stem cells, they are more readily available and easily accessible than other cell sources, such as bone marrow or blood vessels. However, canine ADMSCs have slightly different characteristics from human cells, such as differences in CD marker expression and differentiation potential [14,15]. Nevertheless, we have used allogeneic canine ADMSCs (cADMSCs) as a cell source as they have been shown to have therapeutic effects in various studies [13,16]. In this study, cADMSCs were obtained using a medium supplemented with antioxidants, such as Glutathione (GSH), and low glucose. The homogeneous cells prepared for bone cell treatment were induced towards early bone differentiation for a short period of time and were more suitable for treatment purposes than transplantation of undifferentiated ADMSCs.

Biomaterial scaffolds that provide bone-like microenvironments are commonly used in bone regeneration therapy [17]. Studies have shown that the effects vary depending on the method used to produce the scaffolds and characteristics of the scaffold biochemical composition [18]. Therefore, material selection and fabrication methods are important factors in the development of scaffolds for bone tissue engineering applications, and have a significant impact on the properties of the scaffold [19]. Polycaprolactone (PCL) has the advantages of biocompatibility, biodegradability, low immunity, high mechanical strength, and elasticity, and is widely used in the fabrication of bone tissue engineering scaffolds [7].

Important research tools for evaluating bone regeneration induced by scaffolds implanted into bone defects are X-ray and micro-computed tomography (micro-CT) analysis [20,21]. In particular, micro-CT can display three dimensional (3D) images and characterize the structure of soft tissues to study animals in a non-invasive manner to detect bone abnormalities [22].

The objective of this study was to investigate the regeneration capacity of ADMSCs using an improved biodegradable scaffold method in a canine femoral segmental defect model through X-ray, micro-CT, and histopathology. By measuring bone mineral density (BMD), information on the correct positioning of the scaffold and quantification of bone regeneration was obtained.

## 2. Materials and Methods

### 2.1. Design of the Study

After a 7-day acclimatization period, an animal model of adipose-derived stem cells was used to induce osteogenesis using a biodegradable scaffold. Briefly, surgery was applied to six male beagles. Six dogs were divided into the following groups: (1) control and sham (no treatment), *n* = 3, and (2) cell + scaffold (C/S), *n* = 3. Canine femoral segmental defect was performed, as previously described [23]. Body weight was measured at 1-week intervals and serum was collected at 2-week intervals. After 12 weeks, the animals were sacrificed, and the femurs were isolated.

### 2.2. Primary Cells and Cultures

We obtained approximately 2–3 g of adipose tissue from canines for ADMSC isolation. With owner consent, we obtained fat tissue biopsies from the gluteal region of five young canines at a local hospital. Adipose tissue was cleaned twice with phosphate-buffered saline (PBS) containing 5% penicillin/streptomycin (Thermo Fisher, Gangnam, Seoul, Republic of Korea) to prevent possible contamination. The adipose tissue was then dissected into as small pieces as possible and cells were obtained by reacting with AdiCol™ (CEFO Co., Jong-ro, Seoul, Republic of Korea) with 0.1% collagenase for 30 min at 150 rpm in a 37 °C shaking incubator. The tissue and enzyme were mixed at a 1:1 ratio. Undifferentiated cADMSCs were maintained in CEFOgro™ cMSC (CEFO Co., Jong-ro, Seoul, Republic of Korea) medium with 0.5% penicillin/streptomycin at 37 °C in a 5% CO_2_ humidified incubator. For the induction of osteogenesis, CEFOgro^TM^ DM Osteogenic medium containing 10 mM β-glycerophosphate, 200 µM L-ascorbic acid, and 100 nM dexamethasone was used.

### 2.3. Cell Characterization

We confirmed the expression of cell surface proteins and measured colony-forming units for characterization of cADMSCs. The cells were analyzed by flow cytometry using CD44, CD73, and CD90 antibodies. All antibodies were purchased from BD Biosciences (BD Biosciences, Gangnam, Seoul, Republic of Korea). The cells were washed twice in cold PBS containing 1% bovine serum albumin (BSA) and were then incubated with a saturating concentration of primary antibody for 1 h at 4 °C in a shaking incubator. Cells were washed twice in PBS containing 1% BSA before incubation with secondary antibody for an additional 30 min at 4 °C. Finally, the cells were fixed in 2% paraformaldehyde, and approximately 2000 events were analyzed by flow cytometry (FACScan, BD Biosciences, Gangnam, Seoul, Republic of Korea) using the CellQuest™Prosoftware version 3.2 (Becton Dickinson, Mississauga, ON L5N083, Canada).

### 2.4. Osteogenic Differentiation and Analysis

For osteogenic differentiation, cADMSCs at passage 5 were seeded onto a culture plate (50,000 cells/cm^2^). At 80% confluence, the medium was changed to osteogenic differentiation medium (CEFOgro^TM^ DM Osteo, Seoul, Jong-ro, Republic of Korea) to induce osteogenesis for 24 h. Osteogenic differentiation was analyzed by washing the cells twice with PBS, followed by fixing in 70% ethanol for 10 min at 4 °C and washing three times with distilled water. Alkaline phosphatase (ALP) activity assay was performed to confirm the degree of bone differentiation. The ALP assay kit (GeneTex Inc., Jong-ro, Seoul, Republic of Korea) was performed according to the manufacturer’s protocol. Cell viability was analyzed using an automated fluorescence cell counter (ADAM-MC™, Nano&Tek, Seoul, Guro-gu, Republic of Korea) based on propidium iodide (PI) staining, with advanced image analysis according to the osteodifferentiation period. PI staining and analysis were performed according to the manufacturer’s protocol.

### 2.5. Cell Preparation for Transplantation

**Figure 1:**
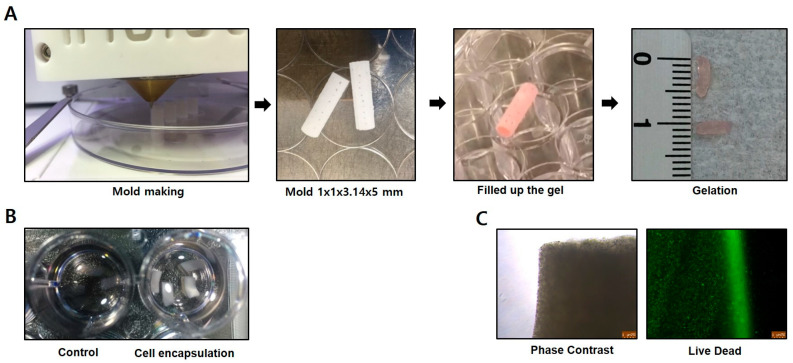
Cell encapsulation for transplantation. (**A**) For encapsulation of the cells, a mold was made with a 3D printer to fit the implant site (diameter: 4.5 mm, height: 20 mm). Cells and gel were mixed to gelation and injected into the mold. Following incubation, the mold was removed, leaving the final product for transplantation (ruler shown for scale). (**B**) The gel was prepared in two types, a gel-only group (sham treatment), and a cell-encapsulated group. (**C**) Cell viability was confirmed by live-dead staining, where live cells are shown in green and dead cells in red.

For transplantation of cells into the long bones of canines, osteogenic-differentiated cells were encapsulation in a hydrogel (4 × 10^6^ cells/capsule). Hydrogels and molds were prepared as follows. First, a PCL-based cylindrical mold (diameter: 4.5 mm, height: 20 mm) was fabricated using a 3D printer (In Vivo Bioprinter, Rokit, Geumcheon-gu, Seoul, Republic of Korea). The mold was then sterilized by precipitation in alcohol for 30 min and air-dried on a clean bench overnight. Second, alginate powder (W201502, Sigma-Aldrich, Jong-ro, Seoul, Republic of Korea) was dissolved in additive-free culture medium at 60 °C for 1 h to prepare a 2.5% pre-gel solution of sodium alginate. Cells were then harvested and centrifuged to obtain pellets. Cells were mixed with the 2.5% pre-gel solution, injected into the PCL-based cylindrical mold, and cross-linked for 15 min using 5% calcium chloride as a cross-linking agent, after which the mold was removed. Cell capsules for transplantation (cross-linked cell-containing hydrogels) were washed three times with additive-free culture medium. At the end of the procedure, the cell capsules were stored in 1 mL of a xeno-free storage solution (CEFO-vive™, CEFO Co., Jong-ro, Seoul, Republic of Korea) at 4 °C until transplantation (Figure 1).

### 2.6. Animals

Six male dogs (Beagle; Woojung BSC, Hwaseong, Gyeonggi-do, Republic of Korea) were used in this study. Animals were maintained in a room at 22 ± 2 °C with a relative humidity of 50 ± 10% and a 12 h light-dark cycle. Animals were fed pellets and water freely. Animal experiments were performed in accordance with the National Institutes of Health guidelines for the care and use of laboratory animals and approved by the Institutional Animal Care and Use Committee of Kyungpook National University, Daegu City, Republic of Korea (KNU-2016-0035) and the Ethics Institute; the date of approval was (19 February 2016), Preclinical Research Center, Daegu-Gyeongbuk Medical Innovation Foundation (DGMIF-20102301-02), Daegu City, Republic of Korea.

### 2.7. Surgical Procedures

**Figure 2:**
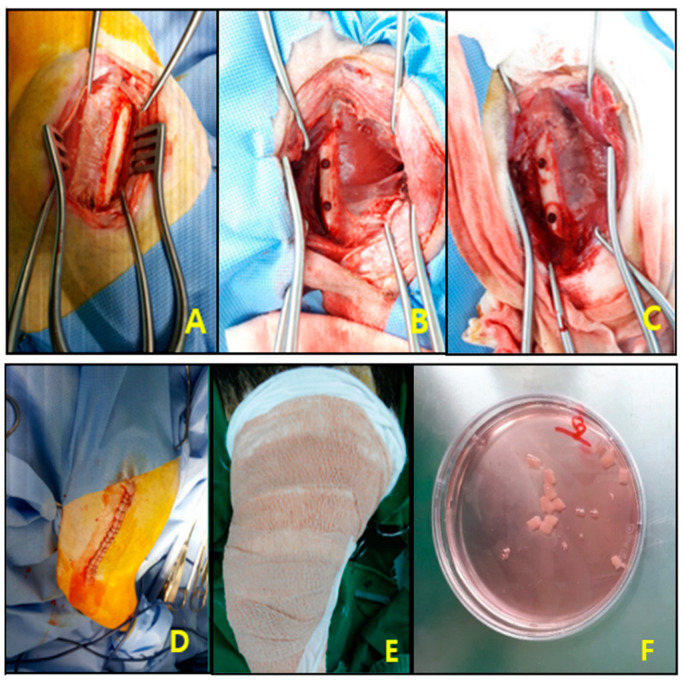
Canine femoral segmental defect model. (**A**) Femur exposure. (**B**) Femur defect (two holes). (**C**) Scaffold transplantation. (**D**) Suture. (**E**) Bandaging. (**F**) Scaffolds prior to transplantation.

To conduct in vivo experiment, a beagle bone defect animal model was surgically established (Figure 2). Beagle dogs were fasted 24 hours before surgery, and a vascular catheter was inserted into the brachial cephalic vein to induce anesthesia using a mixture of alfaxalone (3 mg/kg) and xylazine (2.3 mg/kg). After intubation, respiratory anesthesia was maintained with 2% isoflurane during the cell transplantation. Beagle dogs were placed in the right lateral recumbency position on the operating table, and femur area hair was removed and disinfected. Minimizing soft tissue damage, the skin was incised using a standard approach, the biceps femoris muscle and vastus lateralis muscle were separated, and the femoral diaphysis was exposed using retractors. The periosteum of the femur was separated using a blade, and two bone defects were induced using a surgical drill (Conmed, Hall 50 powered instruments system, Utica, NY, USA) with 5.0 diameter bur at the lower and the upper one thirds of the femur. After hemostasis is achieved, the test material scaffolds were transplanted into the bone damaged areas and covered with a regenerative membrane to prevent fibrosis. Sutures were performed on the muscles with 3-0 Vicryl (J416H, Ethicon Surgical Technologies, Cincinnati, OH, USA), and the skin was sutured with a stapler (Covidien appose ULC skin stapler, Medtronic, Minneapolis, MN, USA). An analgesic (Tramadol, Maritrol, 2.5 mg/kg,) was administered intramuscularly, and the surgical area was disinfected and bandaged using a modified Robert Jones bandage method by 2-inch coban (3M, self-adherent wrap, 1582, tan, Saint Paul, MN, USA) to protect swelling and side effects. Postoperative treatment included analgesics (Tramadol, Tridol, 2 mg/kg, PO) and antibiotics (cefazolin 30 mg/kg, IV until the 3rd day after surgery, and cefadroxil hydrate PO from the 4th to 7th day after surgery) for 7 days after surgery. 

### 2.8. Serum Analysis

A total of 16 serum components were measured using a biochemistry analyzer (TBA-120FR; Toshiba Medical Systems Corporation, Tochigi-ken, Japan) according to the manufacturer’s instructions. The components measured were sodium, potassium, chlorine, total protein, albumin, blood urea nitrogen, creatinine, glucose, total bilirubin, calcium, inorganic phosphate, total cholesterol, triacylglycerol, aspartate aminotransferase, alanine aminotransferase, and alkaline phosphatase.

### 2.9. Imaging Analysis

After surgery, X-ray (ELMO-T3, DK Medical Systems, Seocho-gu, Seoul, Republic of Korea) images of the ventral–dorsal position was captured after 4, 8, and 12 weeks. After necropsy, isolated femurs were imaged using micro-CT (Quantum FX, PerkinElmer, Shelton, CT, USA) according to the manufacturer’s method (parameter: 90 kVp; 180 µA; scan time: 34 s; field of view: 146 mm; threshold: 4000), and BMD was measured.

### 2.10. Histopathology

Harvested femurs were fixed in 10% buffered formalin for one week. Mineralized sections were prepared as follows. After washing with tap water, the samples were dehydrated in a graded series of ethanol (70, 80, 90, 95, and 100%) and infiltrated with a mixture of isopropanol and epoxy resin. They were then embedded in epoxy resin and polymerized in an oven at 60 °C for seven days. The polymerized blocks were cut in the cross direction using a hard-tissue cutting machine (Struer Accutom-50, Büro Ost, Dresden, Germany) under constant cooling. The mineralized sections had an initial thickness of approximately 150 µm and were successively ground to a thickness of approximately 50 µm using a grinding machine (Struer Rotopol-35, Büro Ost, Dresden, Germany). A novel RGB-trichrome staining method was used for tissue staining. Photomicrographs were taken using an Olympus BH2 fluorescence microscope (Olympus Optical Company Ltd., Sinjuku, Tokyo, Japan) equipped with an Olympus DP50 digital camera (Olympus Optical Company Ltd., Sinjuku, Tokyo, Japan). The cortical bone area in the defect was estimated and converted into a percentage of the defect area using image analysis software (ImageJ, National Institute of Health, Bethesda, MD, USA).

### 2.11. Statistical Analysis

We used GraphPad Prism version 6 (Graphpad by Dotmatics, San Diego, CA, USA) for statistical analysis, and statistical significance was determined using the unpaired Student’s *t*-test. The data from each group were expressed as mean ± standard error of the mean, and statistical significance was set at *p <* 0.05 (*), *p <* 0.01 (**), and *p <* 0.001 (***).

## 3. Results

### 3.1. Analysis of Cell Surface Proteins and Osteogenic Differentiation in the cADMSCs

**Figure 3:**
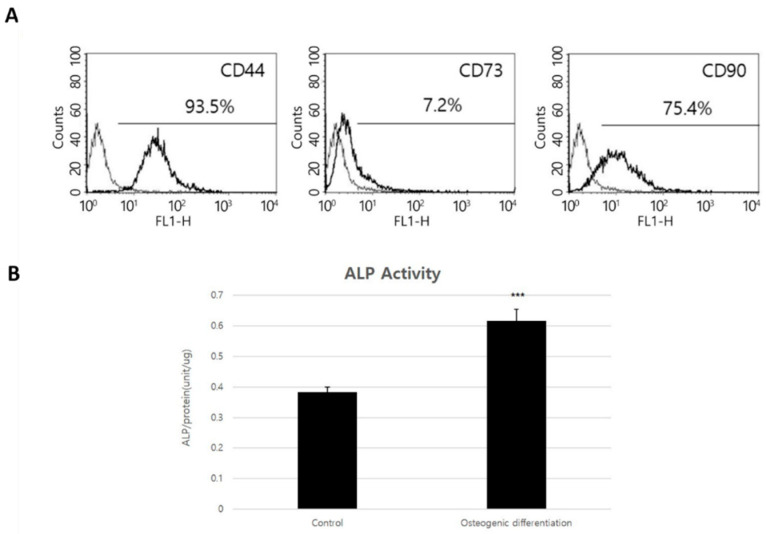
The characterization of stromal vascular fraction (SVF) derived from canine adipose tissue and induction of osteodifferentiation. (**A**) CD protein expression was analyzed in cADMSCs at passage 3 and (**B**) ALP activity after induced osteodifferentiation for 24 h at passage 5 (seeded 50,000 cells/cm^2^), if a *p*-value is less than 0.001 (***).

The cADMSCs expressed CD markers similar to those of typical mesenchymal stem cells, such as CD44, CD73, and CD90 at passage 3 [17,18], but expression of CD73 was very low (Figure 3A). cADMSCs at passage 5 were used to induce osteodifferentiation in animal studies for the treatment of bone defects. Upon investigating the viability of osteodifferentiation-induced cells, it was found that cell viability decreased according to the differentiation induction period. Therefore, early osteodifferentiation was induced for the minimal period of time to increase cell viability. Osteogenic induction of cADMSCs was confirmed by ALP activity analysis. Although osteogenic differentiation was induced for only 24 h, ALP activity was 2 times higher than that of undifferentiated MSCs (Figure 3B). Therefore, osteodifferentiation was induced for only 24 h before the treatment of bone defects, ensuring cell viability was maintained and the early stage of osteodifferentiation had begun.

### 3.2. Body Weight and Serum Analysis

No significant difference in body weight was observed among the groups up to 14 weeks after treatment. In addition, serum biochemistry analysis showed no specific differences among the three groups for the 16 components (Table 1).

### 3.3. X-ray

**Figure 4:**
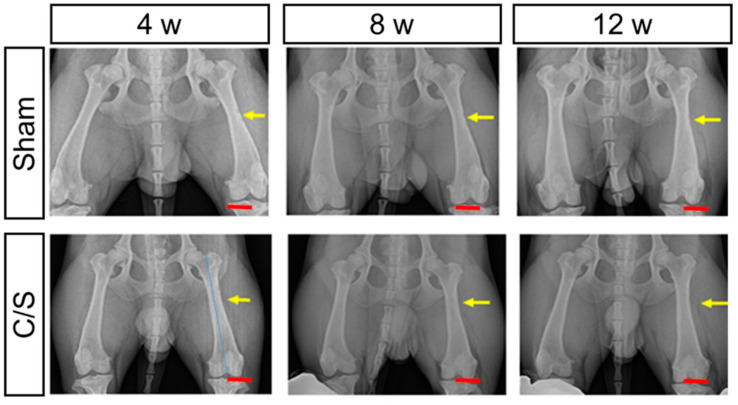
X-ray analysis of the ventral-dorsal position. Right leg was used as the control in each animal. Left leg had either sham, cell + scaffold (C/S). Scale bar = 3 cm. (Yellow line—damaged areas), (Red line—Scale bar).

On radiographic examination, bone regeneration was confirmed 2 weeks after surgery, and the response was clearly observed at 4 weeks. The C/S group showed faster regeneration than the other groups (Figure 4).

### 3.4. Morphology

**Figure 5:**
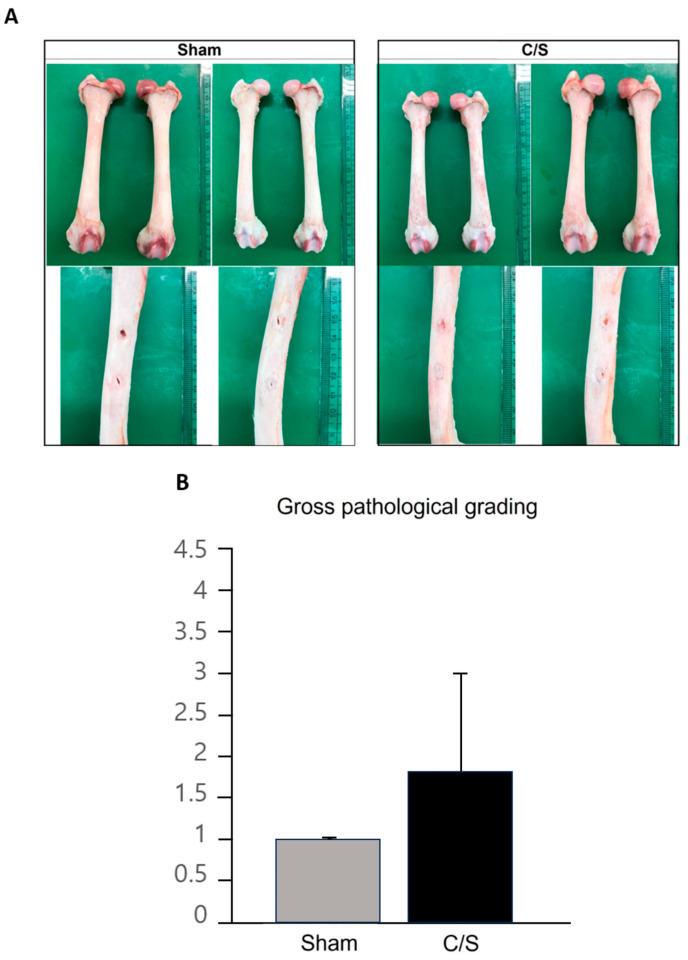
Femoral dissection. (**A**,**B**) The degree of repair of the femur was measured in two separate places for each subject and measured as 1–4 points (1—25%, 2—50%, 3—75%, 4—100%).

Overall, it was confirmed that the degree of restoration was higher than in the sham group (Figure 5A,B).

### 3.5. Micro-CT

**Figure 6:**
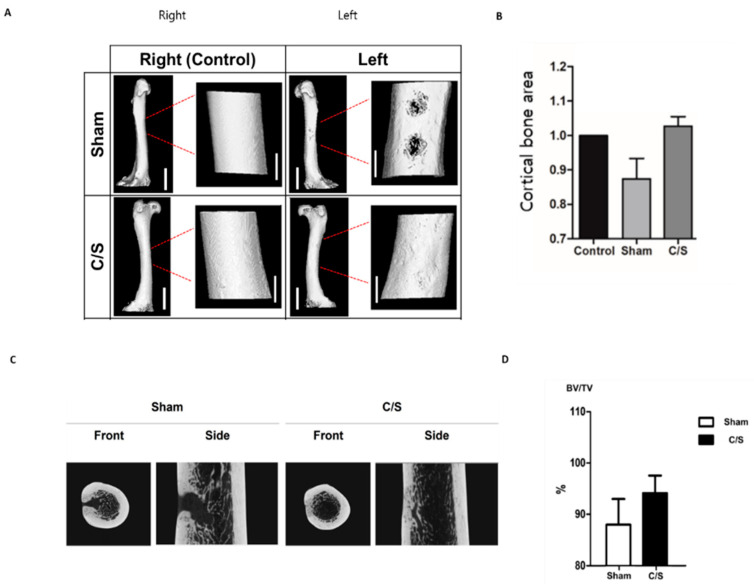
(**A**) Computed tomography(CT) analysis. Right leg was used as the control in each animal. Left leg had either sham, cell + scaffold (C/S). Scale bar = 3 cm in full bone and 1 cm in insert. (**B**) The change in the cortical bone area in the defect area. Bones had no defect (control) or segmental defect with sham, cell + scaffold (C/S). (**C**) Indicates the recovery path of the cortical bone area of the defect area. (**D**) It represents the ratio of the area occupied by bone to the total area. Micro CT analysis, bone volume/tissue volume (mm^3^) measurement, result of the left femur compared to the right femur, the non-surgical control group.

Analysis with micro-CT showed that the degree of bone formation in the defect was greatest in the C/S group, followed by the control groups. The BMD of the defective femur was lower than the control femur in all cases; however, it was not significant in the C/S group (Table 2 and Figure 6A and Figure S1 in Supplementary Materials). Compared to the control group, the change in the cortical bone area in the defect area decreased in the sham group (0.874) whereas it slightly increased in the C/S group (1.027) (Figure 6B). In addition, the C/S group showed a higher degree of bone repair than the sham group (Figure 6C,D).

### 3.6. Histopathology

**Figure 7:**
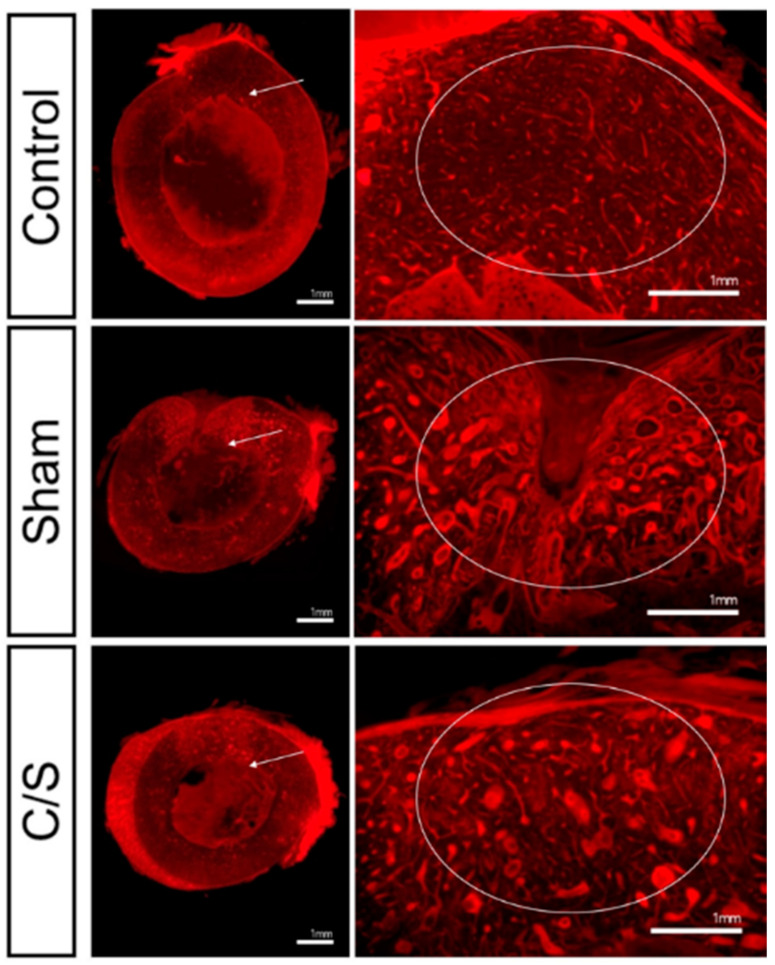
Histopathological analysis following treatment. Bones had no defect (control) or segmental defect with sham, cell + scaffold (C/S). (Original magnification: Left = ×20, Right = ×40). (white arrow-magnification, indicating for the degree of bone regeneration).

In the sham group, although resorption of bone defects was relatively active, the increase in the number of blood vessels and expansion of the surrounding space resulted in a marked decrease in bone density compared to the surrounding normal bone tissue. In addition, a partial depression was observed on the outer surface of the medullary bone, and this region was filled with fibrous connective tissue rather than bone tissue. In contrast, the inner surface of the cusp restored complete continuity, and no specific reactive bone formation was observed. In the C/S group, the area of bone defects recovered to a thickness and shape similar to that of normal coarse bone and did not show a clear difference from the surrounding normal bone. However, the increase in the relative vascular distribution also showed a decrease in overall bone density. A weak depression filled with fibrous tissue was observed on the outer side of the dense bone. The newly formed cortical bone showed a slight difference in bone density compared with the surrounding normal bone tissue as the vascular distribution was increased (Figure 7).

**Figure 8:**
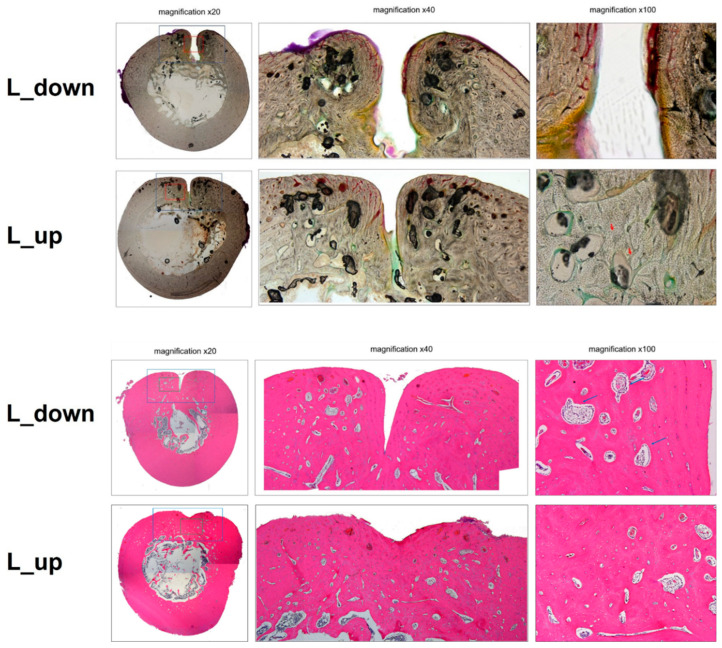
Histopathological inclined bone tissue left leg analysis. Bones had no defect (control) or segmental defect with sham, cell + scaffold (C/S). (Original magnification: ×20, ×40, ×100). Arrows (part of bone structure and lamina propria in dense bone plate), (colorless-Non-exempt organization structure) (Red color-Withdrawal organization).

The gap in the bone defect area was very narrow, it was not filled with complete bone tissue. Large spaces (arrows) containing fibrous bone marrow were observed in the recovered bone tissue. Significant growth of new bone was achieved from the bone defect, but complete osseous union was not achieved. Multiple spaces (arrows) filled with bone marrow tissue were observed in the newly formed bone tissue (Figure 8).

## 4. Discussion

While numerous clinical studies have highlighted the broad clinical potential of mesenchymal stromal cell (MSC) applications, recent experiments have also revealed a significant number of side effects associated with MSC treatment, with thromboembolism and fibrosis being the most commonly reported adverse effects in clinical trials. These side effects are often delayed and dependent on the individual patient’s phenotype, necessitating their recognition during clinical trials and mandatory inclusion in the patient’s informed consent [24]. Both in vivo and in vitro studies have shown that cADMSCs play a central role in bone technology engineering (BTE), providing a variety of novel solutions and applications. Although the use of cADMSCs for regenerative purposes has shown several advantages over other MSCs, their interactions with the microenvironment and the effects of these interactions on differentiation are still unclear. Moreover, although cADMSCs have not been precisely defined owing to their unique differentiation properties, they can be used to identify cells that are best suited for their respective reconstruction purposes. To date, studies on iPSC establishment and MSC differentiation in various animal models are underway, and more stable cells are being established [25,26,27].

Stem-cell-based therapies enhance bone healing by promoting bone formation bioactivity, such as osteogenic differentiation and paracrine signaling [28,29]. This bioactivity is most effective when implanted stem cells receive physical and biochemical support for cell growth, proliferation, nutrient supply, and bone tissue deposition [30,31]. Among the various MSCs that can be extracted from different tissues, ADMSCs have shown promise as suitable treatments for bone regeneration [2,27,32]. Recent advancements in exosomes and microRNAs secreted by MSCs, as well as cell transplantation, hold promise for bone regeneration [9,33,34]. Ongoing research aims to develop treatment protocols for companion animals like dogs, cats, and horses, involving direct injection into stabilized MSCs and specific targeting of microRNAs.

Since fully differentiated cells do not undergo further cell division, the cells used in this study were induced into early differentiation, which only determines the direction of differentiation. The short induction phase before transplantation improved the therapeutic effect compared to undifferentiated stem cells. Additionally, we used a mold to the predicted shape and size of the transplantation site; the cells for transplantation were prepared by encapsulation in a biocompatible hydrogel to match the size of the bone defect, which prevented cell loss and migration to other sites during the in vivo study. However, the role of cells in tissue regeneration remains controversial. Some studies have shown that skeletal regeneration alone is sufficient for bone regeneration [35,36,37]. However, the results of this study showed that groups without ADMSCs showed little bone formation, which was observed at the serrated edges of the surrounding normal bones. In mild bone defects, natural cells with the potential for bone formation migrate into the framework, create new bone tissue, and promote bone repair.

The behavior and osteogenic differentiation of stem cells are significantly influenced by ADMSCs properties via mesenchymal transduction, with osteogenic signals primarily transduced by integrin-linked kinases and focal adhesions. The regulation of osteogenic differentiation has been found to be primarily mediated by mesenchymal stem cells, YAP/TAZ activation through the Ras superfamily Rho GTPases and Rho/ROCK signaling, and ECM-integrin interactions. Additionally, in BMMSCs and periodontal ligament cells displaying corresponding levels of RUNX2 and ALP expression, RUNX2 expression was significantly higher in bone-derived cells and was positively correlated with both matrix stiffness and surface ligand availability [28]. However, if the scaffold is implanted in a defect of critical size, the rate of migration or the amount of native cells from the surrounding tissue may or may not result in a formation that is sufficient to efficiently produce bone tissue before the scaffold breaks down. Studies using BMMSCs for bone regeneration have shown that sparging BMMSCs not only provides a source of osteogenic cells for bone formation, but also secretes growth factors that recruit original cells to the defect [14,23,38]. Cytokines released by canine umbilical cord blood MSCs on the first day after transplantation can also improve bone regeneration [39]. ADMSCs can enhance bone matrix formation under osteogenic conditions and exhibit osteogenic differentiation capabilities [40]. Although comparative research with other cells has not been conducted, some studies have shown that ADMSCs are more stable [41].

The aim of the present study was to investigate the regeneration capacity of adipose-tissue-derived MSCs in improved culture methods on biodegradable scaffolds in canine femoral segmental defect models using radiography and histopathology. Male animals were used as they are considered more suitable for non-metabolic disease models because the estrus cycle of females may have hormonal effects on the disease process. We induced a canine femoral segment defect model and applied ADMSCs to scaffolds in the defect lesions. X-rays were taken at 4-week intervals over a 12-week period, after which the animals were sacrificed and the femurs were collected. Isolated femurs were evaluated by micro-CT and undecalcified histopathologic analysis to quantify bone regeneration, such as increasing BMD and changing cellular morphology.

During the 12-week period, body weight and serum biochemistry showed that early osteogenic-induced cADMSCs after culturing in CEFOgro™ cADMSC did not cause toxicity in the canine bone defect model. The radiological and histological findings confirmed that early osteogenic-induced cADMSCs after culturing in CEFOgro™ cADMSC were biocompatible and did not induce any inflammatory reaction. In addition, areas in the defect sites in the cADMSCs treatment group almost fully recovered to normal tissue. The clinical feasibility and successful translation from bench to bedside require preclinical validation [42,43], and preclinical optimization of biodegradable composite scaffolds with large laboratory animals may facilitate clinical implementation [44]. Research has shown that despite the need for further optimization and understanding of certain factors and pathways, bone tissue engineering constructs utilizing a biomaterial scaffold and MSCs demonstrate great promise as an alternative to conventional bone grafting. The paracrine activity of MSCs via the secretion of cytokines and growth factors plays a crucial role in bone regeneration, promoting direct differentiation into osteogenic progenitors and exhibiting an immunomodulatory effect on effector immune cells, influencing the microenvironment of the damaged tissue [33].

The great advantage of bone regeneration therapy with biodegradable materials is that it does not require a second surgical event for removal, which facilitates rapid recovery [45]. Therapy with biodegradable materials offers unique opportunities to customize both size and structure, enabling them to adapt perfectly to the shape of the bone defect with 3D-reconstructed scaffolds [37,46,47]. As the prevalence of severe bone disorders increases during the aging process, the demand for the development of functional bone grafts will continue to grow. However, current BTE strategies neglect to incorporate the crucial elements necessary for homogenous graft viability, osseointegration, and perusable vascular networks within the graft. To address these limitations, the incorporation of mesenchymal stem cells into bone technology side effects (BTS), acting as a primary source of osteogenic precursors for mineralized bone matrix formation and secreting angiogenic factors to stimulate blood vessel formation, is necessary for optimal vascularization and maintenance of bone tissue [34,48]. Implants can also be developed for patient-specific bone regeneration [9,34,48]. In this study, it was shown that these robust cell tissue engineering models with biodegradable scaffolds provided appropriate biocompatibility and effectiveness. We suggest that cells loaded with biodegradable composites may be promising candidates for further clinical applications in bone regeneration.

## 5. Conclusions

In conclusion, transplanting early osteogenically induced CEFOgro™ cADMSCs improved new bone formation in critical bone defects in canines. Further research is necessary to investigate the application of early differentiation-induced cADMSCs after culturing them in CEFOgro™ cADMSC in various disease models. We confirmed the clinical therapeutic feasibility through pre-clinical conditions for biocompatibility. Biodegradable materials and cell therapy have been used to optimize early-stage bone induction in large laboratory animals, and clinical usage can be expanded. The development of a treatment method using a bioabsorbable therapeutic material is beneficial as defects can be treated early and there is no need for surgery to remove the secondary osseointegration prosthesis. Bioabsorbable therapeutic materials may also be applied to critical areas that are difficult to apply existing integrated materials. This is because the strength of the reaction depends on the shape of the material. In addition, various advantages, such as tissue engineering using 3D images, are expected to improve, showing new possibilities for future treatment technology development and clinical application.

## Figures and Tables

**Table 1:** Serum biochemical analysis in the canine femoral segmental defect model.

Animal	Time	Na	K	Cl	TP	ALB	BUN	CREA	GLU	TBIL	Ca	PHOS	TCHOL	TG	AST	ALT	ALP
		(mmol/L)	(mmol/L)	(mmol/L)	(g/dL)	(g/dL)	(mg/dL)	(mg/dL)	(mg/dL)	(mg/dL)	(mg/dL)	(mg/dL)	(mg/dL)	(mg/dL)	(U/L)	(U/L)	(U/L)
Sham	Pre-operation	148.0	4.4	109.9	6.2	3.2	10.8	0.7	88	0	10.6	6.0	162	25	28	34	132
	2 Weeks	146.8	4.6	109.3	5.7	3.0	15.1	0.6	78	0	10.5	6.7	169	37	26	20	158
	4 Weeks	147.6	4.6	109.7	5.8	3.1	17.1	0.6	77	0	10.6	5.9	176	54	26	23	139
	8 Weeks	147.5	4.6	110.6	6.0	3.3	18.2	0.8	79	0	10.4	6.4	175	33	27	32	119
	16 Weeks	146.8	5.3	111.8	6.3	3.3	27.3	0.8	64	0	10.7	4.4	183	59	29	32	107
C/S	Pre-operation	148.2	5.2	109.2	5.7	3.1	11.6	0.7	94	0	10.4	5.7	185	28	27	33	139
	2 Weeks	147.6	5.2	108.9	6.0	3.1	17.0	0.7	93	0	10.5	6.1	209	76	29	22	237
	4 Weeks	147.0	5.2	107.1	6.2	3.3	18.5	0.7	75	0	10.7	5.6	211	95	31	27	172
	8 Weeks	147.9	5.0	107.7	5.9	3.1	13.3	0.7	67	0	10.1	5.1	200	37	28	31	141
	16 Weeks	147.4	5.4	108.6	6.4	3.1	24.7	0.9	89	0	10.4	5.3	221	98	26	40	157

Segmental defect with sham, C/S: cell + scaffold. Na: sodium. K: potassium. CL: chloride. TP: total protein. ALB: albumin. BUN: blood urea nitrogen. CREA: creatinine. GLU: glucose. TBIL: total bilirubin. Ca: calcium. PHOS: TCHOL: total cholesterol. TG: triglycerides. AST: aspartate aminotransferase. ALT: alanine aminotransferase. ALP: alkaline phosphatase.

**Table 2:** Bone mineral density analysis.

Group	Control/Sham		C/S	
Location	Right	Left	Right	Left
Bone mineral density (mg/cc)	1347.36	1301.57	1361.17	1352.62

Segmental defect with sham, C/S: cell + scaffold.

## Data Availability

Qualified researchers may request data from the Preclinical Research Center, Daegu-Gyeongbuk Medical Innovation Foundation.

## Supplementary Materials

The following are available online at https://www.mdpi.com/article/10.3390/bioengineering10111311/s1, Figure S1: X-ray analysis of the ventral-dorsal position. Right leg was used as the control in each animal. Left leg had either sham, cell + scaffold (C/S). Scale bar = 3 cm.
