# Supplementary material for: Early Osteogenic-Induced Adipose-Derived Stem Cells and Canine Bone Regeneration Potential Analyzed Using Biodegradable Scaffolds
Source: Bioengineering (Basel). 2023 Nov 13;10(11):1311. doi: 10.3390/bioengineering10111311 (PMC10669612; doi:10.3390/bioengineering10111311)
Supplement: Supplementary file 1 [file bioengineering-10-01311-s001.zip › bioengineering-2657710-supplementary.pdf]

bioengineering

Supplementary Materials

Early Osteogenic-Induced Adipose-Derived Stem Cells and Canine Bone Regeneration Potential Analyzed Using Biodegradable Scaffolds

Hyun-Ho Yun [1,2,†], Seong-Gon Kim [2,†], Se-Il Park [3,†], Woori Jo [2], Kyung-Ku Kang [1,2], Eun-Joo Lee [1], Dong-Kyu Kim [2], Hoe-Su Jung [2], Ji-Yoon Son [1], Jae-Min Park [1], Hyun-Sook Park [4], Sunray Lee [4], Hong-In Shin [5], Il-Hwa Hong [6] and Kyu-Shik Jeong [1,7,*]

[1] Department of Veterinary Pathology, College of Veterinary Medicine, Kyungpook National University, Daegu 41566, Republic of Korea; hyun6551@kmedihub.re.kr (H.-H.Y.); qzpmqzpm@naver.com (K.-K.K.); miffy525@hanmail.net (E.-J.L.); jiyoon1095@naver.com (J.-Y.S.); kow612@naver.com (J.-M.P.)

[2] Preclinical Research Center, Daegu-Gyeongbuk Medical Innovation Foundation, Daegu 41061, Republic of Korea; sgkim@kmedihub.re.kr (S.-G.K.); c2dar@kmedihub.re.kr (W.-R.J.); dgkim728@kmedihub.re.kr (D.-K.K.); junghs2000@kmedihub.re.kr (H.-S.J.)

[3] Cardiovascular Product Evaluation Center, Yonsei University College of Medicine, Seoul 14513l, Republic of Korea; seil-park@hanmail.net

[4] Cell Engineering for Origin Research Center, Seoul 03150, Republic of Korea; hsparkkwon@hotmail.com(H.-S.P.); sunray@cefobio.com (S.L.)

[5] Department of Oral Pathology and Regenerative Medicine, School of Dentistry, Kyungpook National University, Daegu 41940, Republic of Korea; hishin@knu.ac.kr

[6] Department of Veterinary Pathology, College of Veterinary Medicine, Gyeongsang National University, Jinju City 52828, Republic of Korea; ihhong@gnu.ac.kr

[7] Institute for Next Generation Unified Technology, Hoseo University, Asan 31499, Republic of Korea

* Correspondence: jeongks@knu.ac.kr; Tel.: +82-53-950-5975; Fax: +82-52-950-5955

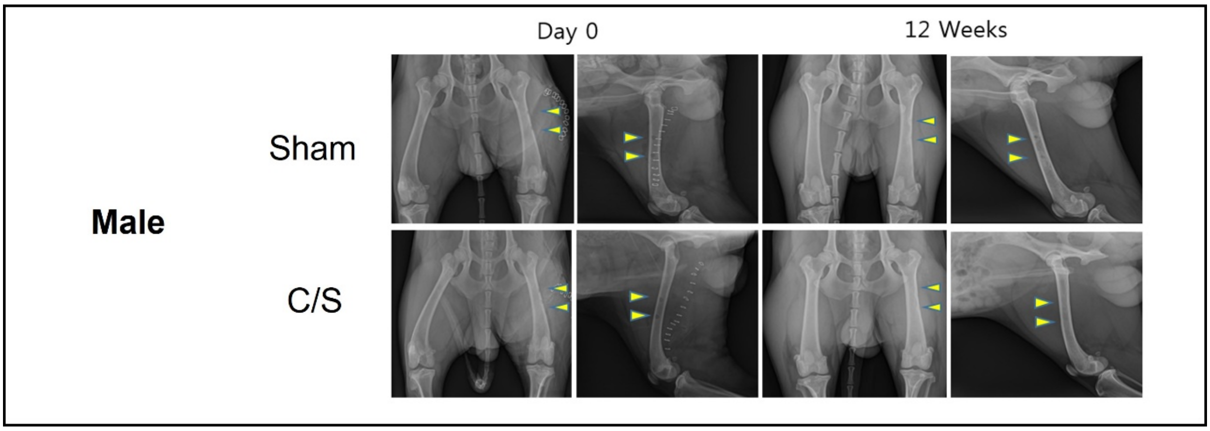

Figure S1. X-ray analysis of the ventral-dorsal position. Right leg was used as the control in each animal. Left leg had either sham, cell + scaffold (C/S). Scale bar = 3 cm.
